# Comparison of clinical outcomes and risk factors for COVID-19 infection in cancer patients without anticancer treatment and noncancer patients

**DOI:** 10.3389/fpubh.2022.925519

**Published:** 2022-08-12

**Authors:** Sen Yang, Huaxin Zhao, Ran Cui, Le Ma, Xuhua Ge, Qiangqiang Fu, Dehua Yu, Xiaomin Niu

**Affiliations:** ^1^Department of General Practice, Yangpu Hospital, School of Medicine, Tongji University, Shanghai, China; ^2^Department of Oncology, Shanghai Tenth People's Hospital, Tongji University School of Medicine, Shanghai, China; ^3^Department of Rheumatology and Immunology, Shanghai Sixth People's Hospital, Shanghai Jiao Tong University, Shanghai, China; ^4^Yangpu Hospital, Tongji University, Shanghai, China; ^5^Department of Shanghai Lung Cancer Center, Shanghai Chest Hospital, Shanghai Jiao Tong University, Shanghai, China

**Keywords:** coronavirus disease 2019, cancer, severe event risk, follow-up, C-reactive protein (CRP)

## Abstract

**Background:**

Previous studies have shown that cancer patients have higher rates of coronavirus disease 2019 (COVID-19) infection and mortality than noncancer patients. However, the differences between cancer patients undergoing regular follow-up without anticancer treatment and noncancer patients with COVID-19 have remained insufficiently investigated.

**Methods:**

A retrospective case–control study of 52 patients with COVID-19 infection was performed with a 1:3 matched proportion of cancer patients undergoing regular follow-up without anticancer treatment and noncancer patients. The demographic characteristics, clinical data, laboratory tests, treatment, and complications of patients were collected from medical records. Chi-square tests and univariate and multivariate regressions were performed to assess the differences between these two cohorts of COVID-19 patients with and without cancer and risk factors for severe events in COVID-19 patients.

**Results:**

Increased C-reactive protein (CRP) (>4 mg/L) (*p* = 0.015) and lactate dehydrogenase (LDH) (>243 IU/L) (*p* = 0.038) were identified as risk factors for severe events in all enrolled COVID-19 patients based on multivariate analysis, but cancer as a chronic disease (*p* = 1.000) was not identified as an independent risk factor for severe events in COVID-19 patients. Compared with noncancer patients, cancer patients had a significantly longer median hospitalization time (29 days vs. 19 days, *p* = 0.048) and a higher incidence of hypoalbuminemia complications (84.6 vs. 46.2%, *p* = 0.016).

**Conclusions:**

Increased CRP and LDH were risk factors for severe events in all enrolled COVID-19 patients, and an increased incidence of hypoalbuminemia complications and longer hospitalization were noted in COVID-19 cancer patients undergoing regular follow-up without anticancer treatment compared with noncancer patients.

## Introduction

The number of people infected by COVID-19 continues to increase with the emergence of many new mutant strains at present (1). Although the pathogenicity has decreased, mutant strains are becoming increasingly contagious, which seriously threatens human health (2, 3). A previous retrospective study showed that 48% (91/191) of 191 COVID-19 patients had multiple chronic diseases at the time they were discharged from the hospital (4). Of note, COVID-19 patients with comorbid chronic diseases, such as hypertension, diabetes, and coronary heart disease (CHD), which have been reported to be the top three chronic diseases (5), are at greater risk for severe events (6).

Currently, cancer patients infected with COVID-19 have received increasing attention. Liang and colleagues (7) showed that COVID-19 patients not receiving anticancer therapy (124/1572, 8%) had a lower risk of severe events than those who had received anticancer therapy (3/4, 75%) in the past month. COVID-19-infected cancer patients who received chemotherapy or surgery within 14 days of diagnosis had faster disease deterioration and worse prognosis than those without anticancer treatment (8). Cancer patients were more susceptible to COVID-19 due to the presence of many underlying diseases, malnutrition, and related side effects of anticancer therapy (9), which could lead to prolonged hospitalizations, severe complications, and even admission to the intensive care unit (ICU) or death (10). Patients who did not require anticancer treatments undergoing follow-up had a lower risk of a severe event (45.7%) than those who had received cancer treatment within the past month (64.9%) (11). Considering that most of the current studies focused on the comparison of COVID-19 patients without and with cancer receiving active cancer treatment, research comparing the impact of COVID-19 between noncancer patients and cancer patients without anticancer treatment has not been reported. This study mainly explored the differences between these two cohorts by comparing the clinical factors and risk factors for severe events between COVID-19-infected cancer patients undergoing regular follow-up and noncancer patients from Wuhan Leishenshan Hospital.

## Materials and methods

### Research protocol design and participants

A total of 52 COVID-19 patients admitted to Wuhan Leishenshan Hospital from February to April 2020 were retrospectively recruited for a 1:3 matched case–control study (12, 13), including 13 patients with different types of cancer undergoing regular follow-up without anticancer treatment irrespective of when the cancer was diagnosed and 39 matched noncancer patients. The following data were obtained from medical records: clinical demographic factors, including sex and age; smoking status; chronic diseases, and treatment. Wuhan Leishenshan Hospital was a designated hospital to treat COVID-19 after the outbreak of COVID-19 in 2020. The research was authorized to be performed by Wuhan Leishenshan Hospital according to Medical and Health Institutions Conducting Clinical Research Projects Management Methods [No. 2014(80)] and was authorized by the Institutional Review Board of Yangpu Hospital Affiliated to Tongji University (No. of ethics approval: LL-2020-KY-003). The diagnosis of COVID-19 followed World Health Organization (WHO) interim guidance (14), and the laboratory diagnosis followed the definition of SARS-CoV-2 infection (15). As previously described (16), nasal and/or throat swabs were obtained to test for SARS-CoV-2 ribonucleic acid (RNA) by reverse transcription-polymerase chain reaction (RT–PCR), which confirmed the presence of COVID-19. To avoid the impact of other chronic diseases, such as hypertension, diabetes, chronic obstructive pulmonary disease (COPD), and CHD, biased factors were balanced in these two cohorts, including the history of chronic disease and other diseases, such as liver damage, kidney damage, and arrhythmia.

### Data collection

Information on participants' demographic characteristics, clinical characteristics, cancer history, laboratory results, treatment options, and clinical outcomes was obtained through electronic medical records and patient interviews. A standardized data collection form modified from the International Severe Acute Respiratory and Emerging Infection Consortium form was used to assess complications (17). All data were independently validated by two medical reviewers. Data including age, sex, smoking status, chronic diseases (diabetes, hypertension, CHD, arrhythmia, cerebrovascular disease, chronic kidney disease, chronic liver disease and anemia), history of malignancy (type of cancer, time of onset and cancer history), vital signs (temperature, respiratory rate, blood pressure, heart rate, and oxygen saturation), symptoms (fever, dry cough, sputum, chest tightness, dyspnea, myalgia, fatigue, anorexia, diarrhea), laboratory results [leukocyte, neutrophil, monocytes, lymphocytes, platelets, albumin, total bilirubin, creatinine, blood urea nitrogen, creatine kinase, lactate dehydrogenase (LDH), D-dimer, prothrombin time, C-reactive protein (CRP), interleukin-6 (IL-6), and interleukin 2 receptor (IL-2R)], COVID-19 treatment [antiviral and antibiotics, corticosteroids, human serum albumin, intravenous immunoglobin, traditional Chinese medicine, renal replacement therapy, convalescent plasma transfusion, and extracorporeal membrane oxygenation (ECMO)] and complications while in the hospital were collected and recorded throughout the diagnosis and treatment process.

### Definitions and outcomes

An axillary temperature exceeding 37.3 °C was defined as fever. The definitions of sepsis and septic shock were in accordance with the third edition of “International Consensus Definitions of Sepsis and Septic Shock” in 2016 (18). Respiratory failure, acute respiratory distress syndrome (ARDS), septic shock, acute cardiac injury, acute kidney injury (AKI), and acute liver injury were defined according to a previous study (19). Activated partial thromboplastin time >3 s or activated partial thrombin activity <5 s is regarded as coagulation dysfunction, and albumin <30 g/L is regarded as hypoalbuminemia (20). The severity status of COVID-19-infected patients was categorized into the following three groups: general, severe, and critical. The diagnosis of severe COVID-19 was confirmed by meeting any one of the following three criteria: respiratory rate >30 times per min, blood oxygen saturation <93% at room temperature, and arterial partial pressure of oxygen (PaO_2_) per fraction of inspired oxygen (FiO_2_) <300 mmHg (6). Patients who met one of the following three conditions were classified as critical cases: respiratory failure requiring invasive ventilation, septic shock, and multiorgan failure (21). Patients who did not meet the diagnosis of severe and critical COVID-19 were defined as general cases. The CURB-65 is an indicator of the severity of community-acquired pneumonia (CAP), and each item is scored as one point for a total of five items, including new-onset disturbance of consciousness, urea nitrogen >7 mmol/L, respiratory rate ≥30 times per min, systolic blood pressure <90 mmHg and/or diastolic blood pressure ≤ 60 mmHg, and age ≥65 years (22). Cancer patients undergoing regular follow-up without requiring anticancer treatment were defined as at least one month from the last treatment (11). Severe events (the composite endpoint) were defined as requiring admission to the ICU, the use of mechanical ventilation, or death (23).

### Statistical analysis

We used the median of the interquartile range (IQR) and *n* (%) to represent continuous variables and categorical variables. Differences between cancer patients undergoing regular follow-up and noncancer patients were compared using the Mann–Whitney *U*-test, chi-square test, or Fisher's exact test. Risk factors associated with a severe event (requiring admission to the ICU, use of mechanical ventilation, or death) were analyzed using univariate and multivariate logistic regression (forward likelihood ratio model) models.

## Results

### Clinical characteristics of COVID-19 patients

A total of 52 COVID-19 patients were recruited from Wuhan Leishenshan Hospital, including 13 cancer patients undergoing regular follow-up (Table 1) and 39 matched noncancer patients. The cancer patients included three patients with lymphoma, two patients with lung cancer, two patients with bladder cancer, and one patient each with liver cancer, thyroid cancer, esophageal cancer, kidney cancer, laryngeal cancer, and colon cancer. The median age was 66 years old (range 54–71) for all patients, 66 years old (range 52–72) for the cancer group, and 66 years old (range 56–71) for the noncancer group. The whole study group included 28 (28/52, 53.8%) male patients and 24 (24/52, 46.2%) female patients, including 8 (8/13, 61.5%) male and 5 (5/13, 38.5%) female patients in the cancer group and 20 (20/39, 51.3%) male and 19 (19/39, 48.7%) female patients in the noncancer group (Table 2). A total of 15 patients (15/52, 28.8%) were classified as severe and critical cases on admission, including 5 cancer patients (5/13, 38.5%) and 10 noncancer patients (10/39, 25.6%). In total, 8 (8/13, 61.5%), 2 (2/13, 15.4%), and 3 (3/13, 23.1%) COVID-19 patients were defined as general, severe and critical cases, respectively, in the cancer group and 29 (29/39, 74.4%), 1 (1/39, 2.6%), and 9 (9/39, 23.1%), respectively, in the noncancer group (Table 2). Most patients had other multiple chronic diseases, including diabetes (9/52, 17.3%), hypertension (26/52, 50.0%), CHD (10/52, 19.2%), arrhythmia (3/52, 5.8%), cerebrovascular disease (7/52, 13.5%), chronic kidney disease (5/52, 9.6%), chronic liver disease (5/52, 9.6%) and anemia (18/52, 34.6%). No differences in age, sex, comorbidities, CURB-65 score, or COVID-19 severity status were noted between these two groups (*p* > 0.05, Table 2). Regarding signs and symptoms, the noncancer patient group had more patients with shortness of breath than the cancer patient group (46.2% vs. 15.4%, *p* = 0.048). Dry cough was the most common symptom with an incidence of 80.8% (42/52, 80.8%) followed by fever (33/52, 63.5%) and shortness of breath (20/52, 38.5%) (Table 2).

**Table 1:** Clinical characteristics and outcomes of cancer patients infected with COVID-19.

	**Sex**	**Age (years)**	**Comorbidities**	**CURB-65 score**	**Time of illness (years)**	**Tumor type**	**COVID-19 severity status**	**Death(yes/no)**	**Requiring admission to the ICU**	**Using of mechanical ventilation**	**Hospitalization (days)**
1	Male	42	Hypertension, Chronic liver disease	0	NA	Lymphoma	General	No	No	No	9
2	Male	47	Diabetes, Chronic liver disease	1	0.3	Lymphoma	Critical	Yes	Yes	Yes	46
3	Female	51	Hypertension	0	NA	Thyroid cancer	General	No	No	No	24
4	Female	53	Hypertension	0	2	Non-Hodgkin lymphoma	General	No	No	No	28
5	Male	65	Anemia	1	NA	Colorectal cancer liver metastasis	General	No	No	No	34
6	Female	66	Diabetes, Anemia	2	0.2	Esophagus cancer	Critical	Yes	Yes	Yes	60
7	Male	66	Hypertension, Diabetes	1	>3	Bladder cancer	Severe	No	No	No	39
8	Female	67	Hypertension, Coronary heart disease, Cerebrovascular disease,Chronic kidney disease	2	13	Kidney cancer	General	No	No	No	10
9	Male	69	Hypertension	1	3	Lung cancer	General	No	No	No	29
10	Female	70	Arrhythmia, Chronic liver disease	1	NA	Lung metastasis of liver cancer	Critical	No	Yes	Yes	44
11	Male	73	Hypertension, Anemia	1	3	Lung cancer	General	No	No	No	19
12	Male	74	Anemia	1	4	Laryngeal cancer	General	No	No	No	45
13	Male	84	Hypertension	1	10	Bladder cancer	Severe	No	No	No	28

**Table 2:** Demographics and baseline characteristics of COVID-19 patients with and without cancer.

	**Total (*n* = 52)**	**Noncancer patients (*n* = 39)**	**Cancer patients (*n* = 13)**	***p-*value**
**Demographics and clinical characteristics**				
Age, years	66 (54–71)	66 (56–71)	66 (52–72)	0.941
**Sex**				0.521
Male	28/52 (53.8%)	20/39 (51.3%)	8/13 (61.5%)	
Female	24/52 (46.2%)	19/39 (48.7%)	5/13 (38.5%)	
**Any comorbidity**				
Diabetes	9/52 (17.3%)	6/39 (15.4%)	3/13 (23.1%)	0.832
Hypertension	26/52 (50.0%)	18/39 (46.2%)	8/13 (61.5%)	0.337
Coronary heart disease	10/52 (19.2%)	9/39 (23.1%)	1/13 (7.7%)	0.416
Arrhythmia	3/52 (5.8%)	2/39 (5.1%)	1/13 (7.7%)	1.000
Cerebrovascular disease	7/52 (13.5%)	6/39 (15.4%)	1/13 (7.7%)	0.815
Chronic kidney disease	5/52 (9.6%)	4/39 (10.3%)	1/13 (7.7%)	1.000
Chronic liver disease	5/52 (9.6%)	2/39 (5.1%)	3/13 (23.1%)	0.093
Anemia	18/52 (34.6%)	14/39 (35.9%)	4/13 (30.8%)	1.000
**CURB−65 score**				0.673
0–1	45/52 (86.5%)	34/39 (87.2%)	11/13 (84.6%)	
2	6/52 (11.5%)	4/39 (10.3%)	2/13 (15.4%)	
3–5	1/52 (1.9%)	1/39 (2.6%)	0/13 (0%)	
**Signs and symptoms**				
Fever (temperature ≥37.3°C)	33/52 (63.5%)	23/39 (59.0%)	10/13 (76.9%)	0.406
Respiratory rate >24 breaths per min	4/52 (7.7%)	3/39 (7.7%)	1/13 (7.7%)	1.000
Pulse ≥120 beats per min	5/52 (9.6%)	5/39 (12.8%)	0/13 (0%)	0.314
Peripheral oxygen saturation ( ≤ 93%)	3/52 (5.8%)	2/39 (5.1%)	1/13 (7.7%)	1.000
Dry cough	42/52 (80.8%)	33/39 (84.6%)	9/13 (69.2%)	0.416
Sputum	6/52 (11.5%)	5/39 (12.8%)	1/13 (7.7%)	1.000
Chest tightness	11/52 (21.2%)	7/39 (17.9%)	4/13 (30.8%)	0.556
Shortness of breath	20/52 (38.5%)	18/39 (46.2%)	2/13 (15.4%)	**0.048**
Myalgia	4/52 (7.7%)	3/39 (7.7%)	1/13 (7.7%)	1.000
Fatigue	12/52 (23.1%)	7/39 (17.9%)	5/13 (38.5%)	0.254
Anorexia	6/52 (11.5%)	4/39 (10.3%)	2/13 (15.4%)	0.632
Diarrhea	5/52 (9.6%)	3/39 (7.7%)	2/13 (15.4%)	0.589
**COVID-19 severity status**				
General	37/52 (71.2%)	29/39 (74.4%)	8/13 (61.5%)	0.596
Severe	3/52 (5.8%)	1/39 (2.6%)	2/13 (15.4%)	0.151
Critical	12/52 (23.1%)	9/39 (23.1%)	3/13 (23.1%)	1.000

*No differences in age, sex, comorbidities, CURB-65 score, COVID-19 severity status or other signs and symptoms (p > 0.05), including fever (T ≥ 37.3 °C), respiratory rate (>24 breaths per min), pulse (≥120 beats per min), peripheral oxygen saturation ( ≤ 93%), dry cough, sputum, chest tightness, myalgia, fatigue, anorexia, and diarrhea were noted with the exception of shortness of breath (p = 0.048). The data are expressed as the median (IQR), n (%), or n/n (%). Difference analysis was performed using the Mann–Whitney U-test, the χ^2^ test, or the Fisher precision test. COVID-19, Coronavirus disease 2019; T, temperature. Bold value indicates that the difference is statistically significant*.

### Results of laboratory tests for COVID-19 patients on admission

Of the 52 patients admitted to the hospital with COVID-19, no differences in leukocyte count (*p* = 1.000), absolute neutrophil count (*p* = 0.665), monocyte count (*p* = 0.704), lymphocyte count (*p* = 0.118), platelet count (*p* = 0.992), albumin (*p* = 0.347), total bilirubin (*p* = 0.358), creatinine (*p* = 0.795), blood urea nitrogen (*p* = 0.261), creatine kinase (*p* = 0.336), lactate dehydrogenase (*p* = 0.894), D-dimer (*p* = 0.812), prothrombin time (*p* = 0.177), CRP (*p* = 0.722), IL-6 (*p* = 0.186), or IL-2R (*p* = 0.862) were noted between cancer patient group and the noncancer patient group (Table 3).

**Table 3:** Laboratory findings for COVID-19 patients with and without cancer on admission.

**Laboratory findings**	**Total (*n* = 52)**	**Noncancer**	**Cancer patients**	***p-*value**
		**patients (*n* = 39)**	**(*n* = 13)**	
Leukocyte count, × 10^9^/L	..	..	..	..
Median (Q1, Q3)	5.9 (4.7–7.0)	5.8 (4.7–7.7)	6.0 (4.5–6.6)	1.000
≤ 10	46/52 (88.5%)	34/39 (87.2%)	11/13 (84.6%)	1.000
>10	6/52 (11.5%)	5/39 (12.8%)	2/13 (15.4%)	..
Absolute Neutrophil Count, × 10^9^/L	..	..	..	..
Median (Q1, Q3)	3.9 (2.7–4.8)	3.9 (2.7–4.7)	3.9 (2.9–4.9)	0.665
Monocyte's count, × 10^9^/L	..	..	..	..
Median (Q1, Q3)	0.5 (0.4–0.6)	0.5 (0.3–0.7)	0.5 (0.5–0.8)	0.704
Lymphocyte count, × 10^9^/L	..	..	..	..
Median (Q1, Q3)	1.2 (0.8–1.6)	1.1 (0.8–1.6)	1.1 (0.6 −1.4)	0.118
<1.0	18/52 (34.6%)	12/39 (30.8%)	6/13 (46.2%)	0.501
≥1.0	34/52 (65.4%)	27/39 (69.2%)	7/13 (53.8%)	..
Platelet count, × 10^9^/L	..	..	..	..
Median (Q1, Q3)	192.0 (155.3–251.5)	194.0 (146.3–284.0)	210.0 (119.5– 291.5)	0.992
<125	6/52 (11.5%)	5/39 (12.8%)	1/13 (7.7%)	1.000
≥125	46/52 (88.5%)	34/39 (87.2%)	12/13 (92.3%)	..
Albumin, g/L	..	..	..	..
Median (Q1, Q3)	35.4 (32.6–38.8)	33.8 (30.7–38.6)	33.1 (28.3–36.0)	0.347
<30	7/51 (13.7%)	4/38 (10.5%)	3/13 (23.1%)	0.504
≥30	44/51 (86.3%)	34/38 (89.5%)	10/13 (76.9%)	..
Total bilirubin, μmol/L	..	..	..	..
Median (Q1, Q3)	10.9 (7.8–13.7)	11.1 (7.4–15.5)	13.4 (11.3 −18.3)	0.358
Creatinine, μmol/L	..	..	..	..
Median (Q1, Q3)	65.0 (53.7–75.6)	66.1 (50.1–86.9)	57.6 (52.7–71.8)	0.795
Blood urea nitrogen, mmol/L	..	..	..	..
Median (Q1, Q3)	5.6 (4.8–6.5)	5.1 (4.6–7.3)	6.2 (4.2–6.6)	0.261
Creatine kinase, IU/L	..	..	..	..
Median (Q1, Q3)	43.0 (31.8–64.5)	40.5 (22.8– 70.8)	50.0 (33.3–79.3)	0.336
Lactate dehydrogenase, IU/L	..	..	..	..
Median (Q1, Q3)	207.5 (170.5 −250.3)	225.0 (199.3 −312.8)	362.5 (190.5–597.8)	0.894
≤ 245	35/49 (71.4%)	27/36 (75%)	8/13 (61.5%)	0.574
>245	14/49 (28.6%)	9/36 (25%)	5/13 (38.5%)	..
D–dimer, mg/L	..	..	..	..
Median (Q1, Q3)	0.9 (0.5–1.9)	1.2 (0.5–2.5)	1.5 (0.4–10.1)	0.812
≤ 0.55	17/47 (36.2%)	12/35 (34.3%)	5/12 (41.7%)	0.912
>0.55	30/47 (63.8%)	23/35 (65.7%)	7/12 (58.3%)	..
Prothrombin time, s	..	..	..	..
Median (Q1, Q3)	11.5 (11.0–12.5)	11.9 (11.4–12.7)	11.7 (11.1 −13.2)	0.177
≤ 13	37/48 (77.1%)	32/37 (86.5%)	7/11 (63.6%)	0.206
>13	11/48 (22.9%)	5/37 (13.5%)	4/11 (36.4%)	..
C–reactive protein, mg/L	..	..	..	..
Median (Q1, Q3)	1.7 (0.5–33.4)	2.4 (0.5–49.0)	21.8 (0.8–54.7)	0.722
<4	10/45 (22.2%)	23/35 (65.7%)	5/10 (50.0%)	0.593
≥4	35/45 (77.8%)	12/35 (34.3%)	5/10 (50.0%)	..
IL−6, pg/ml	..	..	..	..
Median (Q1, Q3)	5.2 (1.5–53.5)	5.4 (1.7–84.8)	37.4 (2.6–1343.7)	0.186
≤ 7	19/33 (57.6%)	16/26 (61.5%)	3/7 (42.9%)	0.422
>7	14/33 (42.4%)	10/26 (38.5%)	4/7 (57.1%)	..
IL−2R, U/ml	..	..	..	..
Median (Q1, Q3)	575.0 (297.5–1358.0)	656.0 (323.5–1283.5)	923.0 (330.8– 2954.0)	0.862
≤ 710	18/31 (58.1%)	13/23 (56.5%)	5/8 (62.5%)	1.000
>710	13/31 (41.9%)	10/23 (43.5%)	3/8 (37.5%)	..

*No significant differences in laboratory findings were noted between these two groups with and without cancer (p > 0.05). Data are the median (IQR), n (%), or n/N (%). Q1 was defined as a 25% confidence interval, and Q3 was defined as a 75% confidence interval; p-values were calculated by the Mann–Whitney U-test, χ^2^ test or Fisher's exact test as appropriate. IL-6, interleukin-6*.

### Treatment for COVID-19 patients

**Figure 1:**
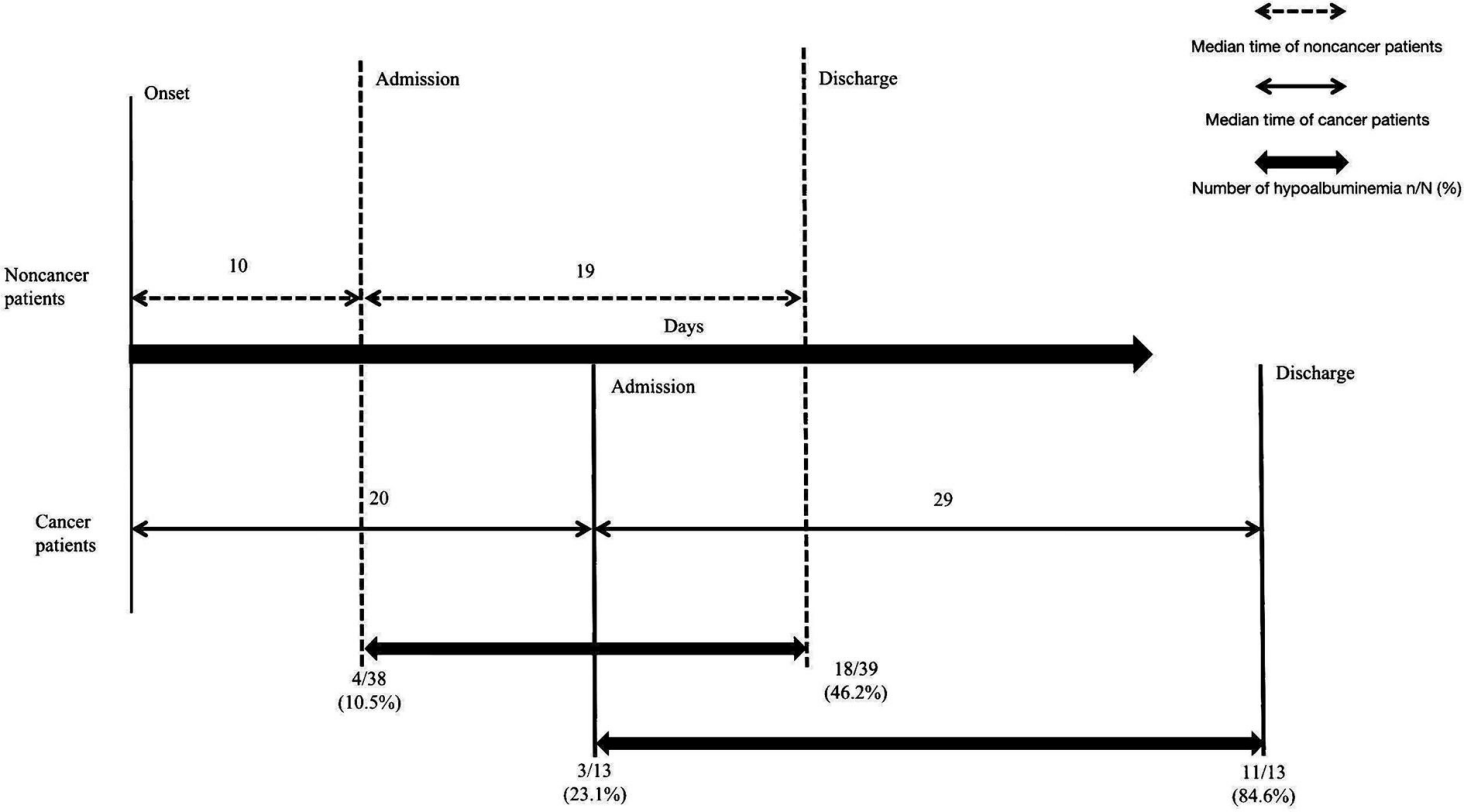
The outcome discrepancy in COVID-19 patients with and without cancer. Compared with noncancer patients, cancer patients experienced a longer time from illness onset to hospital admission (20 days vs. 10 days, *p* = 0.271), a significantly longer median time of hospitalization with treatment (29 days vs. 19 days, *p* = 0.048) and a higher incidence of hypoalbuminemia complications (84.6% vs. 46.2%, *p* = 0.016). Data are expressed as *n* (%), or *n/N* (%).

Regarding treatment for COVID-19 patients, there were 29 patients (29/52, 55.8%) receiving antiviral medications, including 9 cancer patients (9/13, 69.2%) and 20 noncancer patients (20/39, 51.3%, *p* = 0.259). In total, 30 (30/52, 57.7%) patients received intravenous antibiotics, including 8 cancer patients (8/13, 61.5%) and 22 noncancer patients (22/39, 56.4%, *p* = 0.746). In addition, 9 (9/52, 17.3%) patients received intravenous corticosteroids, including 3 cancer patients (3/13, 23.1%) and 6 noncancer patients (6/39, 15.4%, *p* = 0.832). 13 patients (13/52, 25.0%) were administered intravenous human serum albumin, including 4 cancer patients (4/13, 30.8%) and 9 noncancer patients (9/39, 23.1%, *p* = 0.853). In total, 42 (42/52, 80.8%) patients received traditional Chinese medicine, including 9 cancer patients (9/13, 69.2%) and 33 noncancer patients (33/39, 84.6%, *p* = 0.416). Four patients (4/52, 7.7%) received renal replacement therapy, all of whom were noncancer patients (4/39, 10.3%). Six (6/52, 11.5%) patients received convalescent plasma transfusion, including two cancer patients (2/13, 15.4%) and four noncancer patients (4/39, 10.3%, *p* = 0.632). Two patients (2/52, 3.8%) were administered ECMO, including one cancer patient (1/13, 7.7%) and one noncancer patient (1/39, 2.6%, *p* = 0.441) (Table 4). Hypoalbuminemia (29/52, 55.8%) was the most frequent complication followed by acute liver injury (12/52, 23.1%), respiratory failure (7/52, 13.5%), septic shock (7/52, 13.5%), and acute cardiac injury (7/52, 13.5%). Significant differences in the complication of hypoalbuminemia were noted between cancer patients and noncancer patients (84.6 vs. 46.2%, *p* = 0.016, Table 4, Figure 1), but there were no significant differences in the treatment and other complications in patients with and without cancer (Table 4).

**Table 4:** Treatments, complications and outcomes of COVID-19 patients with and without cancer on admission.

	**Total (*n* = 52)**	**Noncancer patients (*n* = 39)**	**Cancer patients (*n* = 13)**	***p*-value**
**Treatments**				
Antiviral treatment	29/52 (55.8%)	20/39 (51.3%)	9/13 (69.2%)	0.259
Antibiotics	30/52 (57.7%)	22/39 (56.4%)	8/13 (61.5%)	0.746
Corticosteroids	9/52 (17.3%)	6/39 (15.4%)	3/13 (23.1%)	0.832
Human serum albumin	13/52 (25.0%)	9/39 (23.1%)	4/13 (30.8%)	0.853
Intravenous immunoglobin	3/52 (5.8%)	2/39 (5.1%)	1/13 (7.7%)	1.000
Traditional Chinese medicine	42/52 (80.8%)	33/39 (84.6%)	9/13 (69.2%)	0.416
Renal replacement therapy	4/52 (7.7%)	4/39 (10.3%)	0/13 (0%)	0.561
Convalescent plasma transfusion	6/52 (11.5%)	4/39 (10.3%)	2/13 (15.4%)	0.632
ECMO	2/52 (3.8%)	1/39 (2.6%)	1/13 (7.7%)	0.441
**Complications**				
Respiratory failure	7/52 (13.5%)	5/39 (12.8%)	2/13 (15.4%)	1.000
ARDS	4/52 (7.7%)	2/39 (5.1%)	2/13 (15.4%)	0.257
Septic shock	7/52 (13.5%)	5/39 (12.8%)	2/13 (15.4%)	1.000
Acute cardiac injury	7/52 (13.5%)	6/39 (15.4%)	1/13 (7.7%)	0.815
Acute kidney injury	4/52 (7.7%)	2/39 (5.1%)	2/13 (15.4%)	0.257
Acute liver injury	12/52 (23.1%)	8/39 (20.5%)	4/13 (30.8%)	0.704
Hypoalbuminemia	29/52 (55.8%)	18/39 (46.2%)	11/13 (84.6%)	**0.016**
**Severe events**				
Requiring admission to the ICU	12/52 (23.1%)	9/39 (23.1%)	3/13 (23.1%)	1.000
Using of mechanical ventilation	10/52 (19.2%)	7/39 (17.9%)	3/13 (23.1%)	1.000
Deaths	6/52 (11.5%)	4/39 (10.3%)	2/13 (15.4%)	0.632
Time from illness onset to hospital admission, days	10 (4-25.3)	10 (4-21)	20 (5.5-28)	0.271
Hospitalization, days	20 (16-33.5)	19 (14-30)	29 (21.5-44.5)	**0.048**

*A longer median time of hospitalization with treatment (29 days vs. 19 days, p = 0.048) and a higher incidence of hypoalbuminemia complications (84.6% vs. 46.2%, p = 0.016) were noted in cancer patients compared with noncancer patients. Data are median (IQR), n (%), or n/N (%). p-values were calculated by the Mann–Whitney U-test, χ^2^ test, or Fisher's exact test, as appropriate. ARDS, acute respiratory distress syndrome; ECMO, extracorporeal membrane oxygenation; ICU, intensive care unit. Bold values indicates that the difference is statistically significant*.

### The outcome discrepancy for COVID-19 patients with and without cancer

The severe events, including admission to the ICU, use of mechanical ventilation, and death, were noted in 12 (12/52, 23.1%), 10 (10/52, 19.2%) and 6 (6/52, 11.5%) patients overall, respectively; 9 (9/39, 23.1%), 7 (7/39, 17.9%) and four patients (4/39, 10.3%) in the noncancer group, respectively; and 3 (3/13, 23.1%), 3 (3/13, 23.1%) and two patients (2/13, 15.4%) in the cancer group, respectively (Table 4). A longer median time of hospitalization with treatment was noted for cancer patients compared with noncancer patients (29 vs. 19 days, *p* = 0.048, Table 4, Figure 1).

### Risk factors for severe events in COVID-19 patients

Univariate and multivariable logistic regression were applied to analyze the risk factors for severe events in all COVID-19 patients. The univariate logistic regression analysis demonstrated that higher CRP (>4 mg/L) [odds ratio (OR) = 18.571, 95% confidence interval (CI): 3.284-105.013, *p* = 0.001], lower albumin (<30 g/L) (OR = 38.000, 95% CI: 3.866–373.524, *p* = 0.002), higher prothrombin time (>13 s) (OR = 11.000, 95% CI: 2.142–56.496, *p* = 0.004), higher LDH (>243 IU/L) (OR = 19.800, 95% CI: 3.958–99.053, *p* < 0.001), higher IL-6 (>7 pg/ml) (OR = 66.000, 95% CI: 6.083–716.151, *p* = 0.001) and higher IL-2R (>710 U/ml) (OR = 93.500, 95% CI: 7.542–1159.073, *p* < 0.001) were related to an increased risk of severe events in all COVID-19 patients, while cancer as a chronic disease itself (OR = 1.000, 95% CI: 0.225-4.436, *p* = 1.000), older age (>65 years) (OR=0.840, 95% CI: 0.226-3.126, *p* = 0.795), and sex (male) (OR = 1.267, 95% CI: 0.344-4.670, *p* = 0.723) were not related to an increased risk of severe events for all COVID-19 patients. Due to the lack of IL-6 data for 19 patients and IL-2R data for 21 patients, CRP, albumin, prothrombin time, and LDH were further included to explore the risk factors for severe events in the multivariable analysis using the forward likelihood ratio model. The results showed that increased CRP (>4 mg/L) (OR = 11.438, 95% CI: 1.607-81.416, *p* = 0.015) and higher LDH (>243 IU/L) (OR = 7.631, 95% CI: 1.123-51.880, *p* = 0.038) were associated with increased risk factors for severe events in all COVID-19 patients (Table 5). Further univariate logistics analysis in all COVID-19 patients showed that cancer as a chronic disease was not identified as an independent risk factor for mortality (OR = 1.591, 95% CI: 0.256–9.894, *p* = 0.619), AKI (OR = 3.364, 95% CI: 0.423–26.718, *p* = 0.251) and requirement of mechanical ventilation (OR = 1.371, 95% CI: 0.298–6.318, *p* = 0.685).

**Table 5:** Risk factors associated with severe events in COVID-19 patients with and without cancer.

	**Univariable OR (95% CI)**	***p* value**	**Multivariable OR (95% CI)**	***p* value**
Cancer as a chronic disease	..	..	..	..
No	1 (ref)	..	..	..
Yes	1.000 (0.225–4.436)	1.000	..	..
Age, years*	..	..	..	..
≤ 65	1 (ref)	..	..	..
>65	0.840 (0.226–3.126)	0.795	..	..
Sex			..	..
Female	1 (ref)		..	..
Male	1.267 (0.344–4.670)	0.723	..	..
Lymphocyte count, × 10^9^/L	..	..	..	..
≥1.1	1 (ref)	..	..	..
<1.1	3.691 (0.965–14.112)	0.056	..	..
Platelet count, × 10^9^/L			..	..
≥125	1 (ref)		..	..
<125	4.111 (0.708–23.855)	0.115		
C–reactive protein, mg/L*		..	..	..
≤ 4	1 (ref)		..	..
>4	18.571 (3.284–105.013)	**0.001**	11.438 (1.607–81.416)	**0.015**
Blood urea nitrogen, mmol/L			..	..
≤ 7.6	1 (ref)		..	..
>7.6	4.375 (0.897–21.336)	0.068	..	..
Albumin, g/L			..	..
≥30	1 (ref)		..	..
<30	38.000 (3.866–373.524)	**0.002**	..	..
Prothrombin time, s			..	..
≤ 13	1 (ref)		..	..
>13	11.000 (2.142–56.496)	**0.004**	..	..
LDH, IU/L			..	..
≤ 243	1 (ref)		..	..
>243	19.800 (3.958–99.053)	**<0.001**	7.631 (1.123–51.880)	**0.038**
IL−6, pg/ml*^a^			..	..
≤ 7	1 (ref)		..	..
>7	66.000 (6.083–716.151)	**0.001**	..	..
IL-2R, U/ml*^b^		..	..	..
≤ 710	1 (ref)		..	..
>710	93.500 (7.542–1159.073)	**<0.001**	..	..

Higher levels of CRP (>4 mg/L) and LDH (>243 IU/L) increased the risk of severe events for all COVID-19 patients in these two cohorts (p < 0.05) according to the multivariate logistic regression model (forward likelihood ratio model). IL-6, interleukin-6; IL-2R, interleukin-2 receptor; OR, odds ratio; CI, confidence interval; LDH, lactate dehydrogenase. ^*^Per 1 unit increase. ^a^19 patients lacked IL-6 data, and 33 patients (33/52, 63.5%) with IL-6 data were included in the univariate analysis. ^b^21 patients lacked IL-2R data, and 31 patients (31/52, 56.9%) with IL-2R data were included in the univariate analysis. Bold values indicates that the difference is statistically significant.

## Discussion

The differences in COVID-19 infection between cancer patients undergoing regular follow-up without anticancer treatment and noncancer patients have not been well studied. This study demonstrated that increased CRP and LDH were risk factors for severe events in all enrolled COVID-19 patients, but cancer as a chronic disease was not a risk factor for severe events in COVID-19 patients. This finding is similar to the findings of a multicenter study in northern London (24). An increased incidence of hypoalbuminemia complications and longer hospitalization was noted for COVID-19 cancer patients compared with noncancer patients.

In our study, CRP, IL-6, and IL-2R were independent risk factors for severe events in all enrolled COVID-19 patients in the univariate analysis, and CRP was an independent risk factor for severe events in the multivariate analysis. CRP, an important opsonin of systemic inflammation and infection severity, could enhance phagocytosis and promote clearance by binding to phosphocholine in the membranes of host cells and pathogens (25). It has been reported that compared with patients with low CRP values, patients with CRP values >5 mg/L have a nearly fivefold risk of developing ARDS (26). In a prior study of 2782 patients hospitalized with COVID-19, CRP concentrations above the median value were associated with AKI, critical illness, and mortality compared with CRP below the median (27), illustrating that CRP can be used as an important marker for the early assessment of COVID-19 severity. IL-6 and IL-2R were independent risk factors for severe events in all COVID-19 patients based on univariate analysis though multivariate analysis of IL-6 and IL-2R could not be performed due to some missing data. Studies have reported that the levels of cytokines (IL-6, IL-2R, and IFN-γ) in cancer patients with COVID-19 are significantly higher than those in noncancer patients (28, 29). A retrospective cohort study of 2052 hospitalized patients with COVID-19 showed that inflammatory factors (highly sensitive CRP, procalcitonin) and cytokines (IL-2R, IL-6, IL-8) were higher in cancer patients compared with noncancer patients (30). SARS-CoV-2 can trigger the production of high levels of proinflammatory mediators, such as IL-6 and IL-1β, which could lead to a cytokine storm (31). One colorectal cancer patient with long-term anti-PD-1 monotherapy developed cytokine release syndrome (CRS) 5 days after receiving the Pfizer-BioNTech mRNA COVID-19 vaccine with evidence of increased inflammatory markers and elevated cytokine levels (IFN-γ, IL-2R, IL-18, IL-16, and IL-10) (32).

Elevated LDH levels in COVID-19 patients are associated with severe events (33). In a case–control study including 124 patients with severe COVID-19 on admission, LDH (>481 U/L) was an independent risk factor associated with death events (34). A similar result was obtained by setting the cutoff point at the upper limit of the normal value for LDH (243 U/L) in our study. According to a meta-analysis including 3117 hospitalized COVID-19 patients, the mean value of LDH in severe patients was increased 1.54-fold compared with that in non-severe cases (35). In addition, elevated baseline LDH levels were significantly associated with risks of ARDS and mortality (36). The results of these studies were consistent with ours, suggesting that LDH could be considered an independent predictive laboratory value for assessing the severity of COVID-19.

Hypoalbuminemia in severe COVID-19 cancer patients has been repeatedly addressed in the literature (37, 38). In our study, we found that the incidence of hypoalbuminemia complications in COVID-19 cancer patients was greater than that in noncancer patients. This result is consistent with a study in North London, in which COVID-19 patients with cancer were more likely to have hypoalbuminemia (40% vs. 23%, *p* = 0.07) than noncancer patients (24). Hypoalbuminemia has been demonstrated to be associated with lower survival among 121 cancer patients with COVID-19 in a prospective study in Mexico (39). Albumin is a response to the body's nutritional level, and cancer patients may have worse protein synthesis, increased energy consumption, and a slower recovery, which may explain the longer hospitalization duration compared with noncancer patients. Therefore, adequate protein supplementation is indispensable in COVID-19 cancer patients, especially when patients have severe COVID-19.

There are some limitations to our research. First and foremost, this study included different types of cancer patients, so heterogeneity could not be avoided. Second, all the participants in our cohort were from local hospitals, which cannot represent all the profiles of the entire nation. Finally, more detailed information about previous anticancer treatments for the cancer patients in our study could not be obtained, which may have an impact on further analysis. Therefore, larger sample sizes and multicenter studies are needed in the future to explore the differences between COVID-19 patients with and without cancer.

## Conclusion

In summary, our study showed that increased CRP and LDH were risk factors for severe events in all enrolled COVID-19 patients, and there was an increased incidence of hypoalbuminemia complications and longer hospitalization for COVID-19 cancer patients without anticancer treatment undergoing regular follow-up compared with noncancer patients.

## Data availability statement

The original contributions presented in the study are included in the article/supplementary material, further inquiries can be directed to the corresponding author/s.

## Ethics statement

The studies involving human participants were reviewed and approved by Wuhan Leishenshan Hospital according to Medical and Health Institutions Conducting Clinical Research Projects Management Methods [No. 2014(80)], and was authorized by the Institutional Review Board of Yangpu Hospital Affiliated to Tongji University (No. of ethics approval: LL-2020-KY-003). The research content complied with the Declaration of Helsinki, and informed consent was obtained from all individual participants included in the study. The patients/participants provided their written informed consent to participate in this study.

## Author contributions

XN and SY conceptualized and designed the study, performed the analyses, drafted the initial manuscript, and reviewed and revised the manuscript. SY, HZ, RC, and LM designed the data acquisition templates and collected the data. SY, HZ, XG, and QF performed the initial analyses. DY supervised data collection and critically reviewed the manuscript. All authors approved the final manuscript as submitted.

## Funding

This project was supported by grants from Shanghai Sailing Program (No. 20YF1444900), Shanghai Hospital Association Hospital Management Research Fund (No. Q2020049), Project in Yangpu Hospital Affiliated to Tongji University (No. Se1201924), Advanced Appropriate Technology Promotion Project of Shanghai Municipal Public Health Bureau (No. 2019SY048), Beijing Xisike (CSCO) Clinical Oncology Research Foundation (No. Y-HS202101-0205), Guangdong Association of Clinical Trials (GACT)/Chinese Thoracic Oncology Group (CTONG), and Guangdong Provincial Key Lab of Translational Medicine in Lung Cancer (CTONG-YC20210103).

## Conflict of interest

The authors declare that the research was conducted in the absence of any commercial or financial relationships that could be construed as a potential conflict of interest.

## Publisher's note

All claims expressed in this article are solely those of the authors and do not necessarily represent those of their affiliated organizations, or those of the publisher, the editors and the reviewers. Any product that may be evaluated in this article, or claim that may be made by its manufacturer, is not guaranteed or endorsed by the publisher.

## Abbreviations

COVID-19: Coronavirus disease 2019
SARS-CoV-2: Severe acute respiratory syndrome coronavirus 2
CRP: C-reactive protein
ICU: Intensive care unit
RT–PCR: Reverse transcription-polymerase chain reaction
COPD: Chronic obstructive pulmonary disease
CHD: Coronary heart disease
IL-6: Interleukin-6
IL-2R: Interleukin 2 receptor
AHF: Acute heart failure
AKI: Acute kidney injury
IQR: Interquartile range
ARDS: Acute respiratory distress syndrome
OR: Odds Ratio
95% CI: 95% confidence interval
ACE2: Angiotensin-converting enzyme II
ECMO: Extracorporeal membrane oxygenation
NA: Not available.
